# Online accounts of gene expression profiling in early‐stage breast cancer: Interpreting genomic testing for chemotherapy decision making

**DOI:** 10.1111/hex.12832

**Published:** 2018-11-01

**Authors:** Emily Ross, Julia Swallow, Anne Kerr, Sarah Cunningham‐Burley

**Affiliations:** ^1^ The Usher Institute University of Edinburgh Edinburgh UK; ^2^ School of Sociology and Social Policy University of Leeds Leeds UK

**Keywords:** breast cancer, chemotherapy, decision making, gene expression profiling, internet research, sociology

## Abstract

**Background:**

Genomic techniques are being developed within oncology and beginning to be experienced within routine cancer care. Little is known about how these tools feature in patients’ experiences of treatment decision making.

**Objective:**

This research explores the ways in which women interpret and discuss gene expression profiling for breast cancer treatment decision making, as articulated within online accounts.

**Design:**

This study used a qualitative approach to analyse written exchanges focusing on gene expression profiling in the UK (Onco*type* DX test). Accounts are taken from online forums hosted by two UK cancer charity websites, comprising 132 discussion threads from a total of seven forums. Authors qualitatively analysed the data and developed key themes drawing on existing literature from medical sociology.

**Findings:**

Women used online spaces to share and discuss results of gene expression profiling. Women interpreted results in the context of indirect experience of cancer treatment, and sociocultural depictions of cancer and chemotherapy. Users largely represented the test positively, emphasizing its ability to “personalize” treatment pathways, though many also pointed to inherent uncertainties with regards the possibility of cancer recurrence.

**Discussion and Conclusions:**

We highlight the complex contexts in which genomic techniques are experienced, with these shaped by personal biographies, online environments and pervasive cultural narratives of cancer and its treatment. We highlight tensions between the claims of genomic testing to aid treatment decision making and patient reflections on the capability of these techniques to resolve uncertainties surrounding treatment decisions.

## 1 INTRODUCTION

A key feature of contemporary medicine is the incorporation of molecular information within clinical pathways to understand and act upon disease.^1^ This is particularly visible within oncology, where it is anticipated that the identification of specific genetic alterations within tumours will lead to treatment regimes tailored to individual patients.^2^ As an example of this novel approach, gene expression profiling may now be offered as part of routine National Health Service (NHS) breast cancer care for a subgroup of patients. This technique is used to assist decision making around adjuvant chemotherapy, a treatment administered following surgery to reduce the likelihood of cancer returning. For some early‐stage breast cancers,^*^ the predicted benefit of chemotherapy for preventing recurrence may be unclear when assessed on protein receptor status and tumour grade alone. Available as part of NHS care as of April 2015 (initially in England), the Onco*type* DX test uses gene expression profiling to predict the risk of cancer recurrence in these patients and identify those who are most likely to benefit from adjuvant chemotherapy.

Onco*type* DX assesses the activity of 21 genes in breast cancer tissue. The corresponding results are prognostic, indicating the likelihood of a woman's cancer returning within 10 years when treated with hormone therapy alone. The cancer is assigned a continuous “recurrence score” (from 0 to 100), and a risk category for recurrence: low (<18), intermediate (18‐30) or high (≥31).^3^ The recurrence score is a predictor of benefit from the addition of chemotherapy to hormone therapy for disease‐free survival.^4^ In clinical practice, both the score and risk categorization are used by clinicians and patients to assist chemotherapy decisions. For those positioned at low risk of recurrence, studies have suggested that these patients are unlikely to derive great benefit from adjuvant treatment.^5^ Patients in this category are not recommended to proceed with chemotherapy,^6^ which is itself associated with (sometimes severe) side‐effects and suffering.^7^ Chemotherapy is recommended for those patients with a high recurrence score, as it has been shown to bestow significant advantage for disease‐free survival compared with hormone therapy alone.^4^ For those placed in the intermediate category, recommendations for chemotherapy are less clear (though a recent clinical trial has indicated that women with an intermediate score may be spared chemotherapy^8^). In the case of an intermediate score, treatment recommendations often involve further discussions with the patient, alongside consideration of wider clinical parameters and patient preferences.^6^, ^9^

In 2013, guidance published by the National Institute for Health and Care Excellence (NICE) acknowledged the uncertainties surrounding treatment decision making for patients with early‐stage breast cancer of this type and recommended that Onco*type* DX be adopted by the NHS:

Breast cancer patients face significant emotional and psychological strain when considering chemotherapy. It can be particularly distressing for patients in whom the decision to have chemotherapy is unclear… Tools or tests that help people decide whether or not to have chemotherapy are likely to be greatly appreciated by patients.^3^

Here, the availability of gene expression profiling is positioned as a positive development for individual patients, by “helping people to decide” whether to proceed to chemotherapy. Discerning who may (not) benefit most from chemotherapy is also important from a policy perspective, with the “over‐treatment” of breast cancer having implications for health service costs and delivery.^3^, ^10^ Though social scientific research has explored clinicians’ experiences of interpreting gene expression tests^9^ and their impact on professional roles and identity,^11^ less attention has been given to the ways in which these tools feature in *patient* decision making with regards treatment, or the role they play in experiences of cancer more widely. This is important for policy and practice; it has been established that chemotherapy decisions are shaped by social contexts, familial relationships and wider health histories, but little is known about how novel prognostic techniques intersect with these. In this article, we explore some of these factors as articulated by women within online accounts.

Existing sociological research has shown that medical decision making by patients is complex and situated. Treatment decisions may be thought of as “distributed”^12^—shared amongst patients, their families, clinicians and wider social networks, and as occurring across time and space.^13^ Although patient participation in decision making is advocated within medical practice, it has been reported that patients vary in the degree to which they wish to take full ownership over treatment decisions in health care.^14^, ^15^ Alongside contemporary shifts in the provision of care, individuals are also seeking advice and support for medical decision making beyond the clinic through virtual platforms.^16^ The Internet can be a source of second opinions, advice regarding symptoms or side‐effects, and information about tests and treatments for those experiencing cancer.^17^ Indeed, access to others’ experiences of a shared health condition has been highlighted as a key aspect of online information seeking, with first person accounts of illness shaping treatment choices and the very experiences of ill health.^18^ As categorizations of (some) cancers and treatment pathways become more diffuse, patients are today presented with large amounts of information about their condition and a range of different options, including novel therapies and clinical trial participation. In this context, insight into other patients’ experiences and choices may be more salient, as individuals become more active in choosing treatments and options for long‐term management.

To improve understanding of how novel techniques might be shaping patient decision making, in what follows we explore women's experiences of gene expression profiling (Onco*type* DX) in early‐stage breast cancer, as discussed within postings on UK online forums.

## 2 METHODS

### 2.1 Rationale

The online research presented here took place alongside qualitative interviews for a wider study exploring experiences of genomic techniques within contemporary oncology research and practice. The design of the research has been informed by members of two patient and public involvement (PPI) panels, who have raised predictive and diagnostic genomic testing as a topic of concern. Conversations with panel members suggested that these tests may produce false negative/positive or inconclusive results and indicated that uncertainties inherent within genomic testing should be discussed with patients and their families. As a recent introduction to NHS management of breast cancer, and with scant qualitative exploration of patient experiences, Onco*type* DX testing was identified as a relevant technique through which to explore such issues within the remit of the wider research project.

The selection of online forums to access experiences of this healthcare technology was also informed by meetings with PPI panel members. Members have discussed the issue of diversity in experiences of cancer care, and the difficulties that may be faced by those who are “socially excluded” in articulating questions or complaints about care with health professionals. Online forum data provide access to such reflections, with Internet communication often used by individuals to follow‐up clinical diagnoses and compare clinical information with other users.^18^ Further, online forums provide access to a range of experiences from a large number of geographically diverse individuals, who may be excluded from face‐to‐face forms of qualitative research due to disability or their omission from opportunities to participate.^19^ The method also responds to a recognition within the social sciences that Internet use is enmeshed with contemporary experiences of health and illness.^16^, ^20^ Indeed, as landscapes of disease categorization and treatment shift in line with advances in prognostic testing and therapies, decision making in cancer care is becoming more complex.^21^ This has implications for online spaces and their functions, with these subject to ongoing reconfiguration.

### 2.2 Data

To gather online accounts of women's experiences, two authors (ER and JS) searched for the term “Oncotype” within publically accessible online forums, hosted by two UK cancer charity websites: one supporting all cancer types and the other a breast cancer charity. Posts included in analysis were limited to those authored from April 2015, when the test was approved for NHS use, until May 2017. Discussion threads identified by the search were copied into Microsoft Word documents to facilitate qualitative analysis. This application was favoured over Computer Assisted Qualitative Data Analysis Software (CAQDAS) to enable easier movement between analytic memos, which were recorded alongside the text itself using the “comments” function.

Searches yielded a large amount of data. By way of example, one of the seven online forums featuring the word “Oncotype” contained 68 threads within the date range. Discussion threads on this forum contained between 4 and 75 individual posts. To generate a manageable dataset, data from two forums on the breast cancer charity website were excluded from analysis. These two forums focused on (a) experiences of recent diagnosis and (b) discussion of more general topics beyond cancer. Threads where Onco*type* DX did not form the substantive content of discussion were excluded from the recent diagnosis forum, for example if the technique was merely named when recounting treatment pathways. The largest threads excluded here were from women seeking emotional support for specific aspects of their cancer experiences, for example questioning whether their emotions were “normal” (137 pages), and when feeling “low” (118 pages). A total of 32 threads, comprising 950 pages, were excluded, as well as an additional ongoing thread containing over 5000 posts; this also centred around support in the context of recent diagnosis. In the general discussion board, a total of two threads were excluded comprising 433 pages. The first was a thread welcoming users to the forum, and the second concerned a user seeking advice with regards her mother's cancer treatment.

Included threads encompassed comments from those who had not undergone the test themselves, but who had outlined anecdotal or media reported information about the Onco*type* DX test, and from those who had unsuccessfully attempted to access gene expression profiling. From the cancer‐wide website, 53 discussion threads from one forum were taken forward to analysis, and from the breast cancer charity website, 79 threads from six forums were included. This gave a final dataset of 132 discussion threads, comprising 639 pages.

### 2.3 Data analysis

Analysis took a thematic approach, aligned with the analytic process described by Braun and Clarke.^22^ The content of entire discussion threads selected for analysis was read by each author conducting the search, who “constantly compared”^23^ the text within and between threads. This was performed with reference to existing sociological literature on cancer illness narratives, biomedicalization and treatment decision making. This process was also informed by ongoing PPI activities, during which differences in individual patients’ desires to engage with clinical information have been emphasized, as have the uncertainties faced by patients when given information about diagnosis and prognosis. With this literature and PPI insight in mind, ER and JS each developed key themes from their set of data, which were shared electronically and deliberated during several in‐person meetings. Each author then examined both sets of themes and associated extracts, and grouped relevant quotes and concepts within refined key themes. These were then shared between all authors and discussed and developed drawing on verbatim data, to cultivate overarching focal points for the presentation of findings.

### 2.4 Ethical considerations

Online methods of data collection for sociological research are subject to wider ethical guidelines associated with the discipline, with safeguarding the interests of those involved in or affected by the research remaining paramount.^24^ However, the use of online material demands new ethical considerations, with issues of informed consent, anonymity and confidentiality not adequately addressed by guidelines applied to more established research methods. In line with the deliberative process advocated by the Association of Internet Researchers,^25^ we consulted existing online research studies when deciding upon our strategy. Due to the personal nature of the accounts presented within online forums, we contacted forum moderators in May 2017 seeking permission to use individual posts in our research, which was granted. Our approach to the use of online data was approved by departmental Research Ethics Committees at the University of Edinburgh and University of Leeds. To ensure anonymity, as far as is possible with online research, we have assigned updated pseudonyms to users and excluded identifying information in the findings reported below. Dates of individual posts are included to demonstrate the ongoing nature of contributions by individual authors and continued resonance of key themes over time.

## 3 FINDINGS

In what follows, we show how the Onco*type* DX test was represented by women using online forum spaces. We then go on to discuss how women described test results and their role in chemotherapy decision making, with this shaped by indirect experiences of cancer treatment, and sociocultural depictions of cancer and chemotherapy. Finally, we outline how users discussed the test in relation to inherent uncertainties with regards the success of treatment and possibility of cancer recurrence. In doing so, we highlight tensions between the claims of genomic testing to aid treatment decision making, and the uncertainties and anxieties which the procedure could provoke or leave unresolved.

### 3.1 Representations of gene expression testing within online environments

Many discussions of Onco*type* DX represented the test as facilitating treatment decision making, echoing the rationale outlined within the 2013 NICE guidance cited above. For example, one user of a cancer support charity forum explained that gene expression profiling “help[s] make informed decisions about chemo[therapy]” (*DiamondMary, Jan 2017*), and another declared she was “glad I took the test because it did help my decision in the end” (*SandyP, Jan 2017*).

Related to this, several users emphasized the test's ability to provide “personalized” information about cancer. For example, one woman advised others to undergo the test because “it's based on you and you can then make an informed decision” (*Stacey1954, Jan 2016)*. Another described that “what it does is show whether your specific tumour cells would benefit from chemo” (*Telophene, Jun 2017)*. Due to the ability to provide what many discussed as a “specific” prediction of cancer recurrence, some represented Onco*type* DX as providing certainty:

The onco[type DX] test is a very good test, which provides statistical evidence based upon your tumour. It's tailor made and was designed so that it takes the guess work out of whether you will benefit. DiamondMary, May 2016

In expressing its ability to provide “tailor made” information, some juxtaposed gene expression profiling with other sources of information used by clinicians to predict the benefit of adjuvant chemotherapy. This included the NHS *PREDICT* tool, an algorithm used by oncologists to guide chemotherapy decision making. In addition to being used by clinicians, this can be freely accessed by patients online. One user noted that although the Onco*type* DX is “not infallible,” it is preferable to:

the original %s which were based on averages of patients with similar diagnosis […] [Onco*type* DX] actually tests your tumour so it is only based on your data […] it is a lot more personal and specific. Lizdene, Jun 2016

Scepticism of these more widely used tools was also visible amongst others, with *MayP (January 2017)* describing these as “only a general indicator based on past recovery data that can be quite old.” In the same thread, *Pumpkin* noted that where “generalised tools could be very wrong,” Onco*type* DX is “an individual test for you,” which she interpreted as providing more surety with regards her treatment decision.

*Betty45*, who had not experienced gene expression profiling herself but was living with a heart condition she attributed to chemotherapy, displayed a particularly positive view of the technology. She invoked a hopeful future by saying “I think the answer for a large percentage of us will be the Onco*type* DX test which shows whether chemo will work or not” (*Nov 2015*). Some thus positioned the test as not only providing certainty, but also as having the potential to transform breast cancer care.^26^, ^27^ Although this was not reflective of all users, with many also depicting the test not as a tool that eliminated guesswork, but one that “refined” guesswork, positive reflections on the test's role in decision making were visible throughout forum posts. In many cases, these could efface the complex and relational contexts within which decision making arose, which were also articulated within forum discussions. This is discussed further below.

### 3.2 Gene expression testing and treatment decision making

Onco*type* DX's production of a single figure to indicate recurrence risk, and corresponding recommendation to proceed (or not) to chemotherapy, was welcomed by many women. Some attributed a particular power to the test score in shaping their treatment choices. This was evident in reflections on the implications of forthcoming results. For example, two users declared:

[…]if it came back as high risk I would have to [undergo chemotherapy] KyliePear, May 2016

I know if my score is high then I cannot refuse [chemotherapy]. HazelKew, April 2017

Some users stressed Onco*type* DX results when advising those who were unsure about whether to proceed to chemotherapy. As one woman noted, “[the score] suggested chemo would be of benefit to you. Can you really afford to go against that?” (*DiamondMary, March 2017*). The power and influence attributed by some to the test result in treatment decision making were perhaps most evident in representations of test results in the context of *not* proceeding to chemotherapy. For those who had received a low risk score and not proceeded to adjuvant chemotherapy, test results were often depicted as determining this decision, seen in the extracts below:

Had oncotype [DX] score of 15 which means no chemotherapy HeidiD, Jan 2017

I have [an] Oncotype DX score of 17 so no chemo MollyC, Feb 2017

These crude presentations of treatment pathways were common on the forum posts we analysed. Here, we see that uncertainties surrounding the prediction of recurrence risk, and complexities of treatment decision making visible within other forum responses, and described within existing sociological literature [eg, 12,13], were obscured. These users depicted their decision not to go ahead with chemotherapy as fully predicated on their gene expression profiling test result. By describing chemotherapy choices in this way, online accounts of gene expression results suppressed the uncertainties inherent within (recurrence) risk prediction, instead presenting test scores as a “hard reality” inspiring (in)action and emotional responses.^28^ For one woman, a score placing her at “high risk” of recurrence led her to defy a personal preference to avoid chemotherapy, for which she had previously “fought”:

I had the Onco*type* test and got a score of 47 so it was a very easy decision for me. (Until I got the result I was fighting tooth and nail to avoid chemo, but I listened to the data.) CancerBeater, Jul 2016

As seen above, presentations of the test score as authoritative were particularly evident amongst women attaining a low score, who often portrayed their chemotherapy choice as unambiguous (though of course, this was not necessarily experienced as such). In contrast, women attaining intermediate (and to a lesser extent high) scores generally described treatment decision making following Onco*type* DX as more complex, fraught and fragmented within their forum posts. Unlike high and low designations of recurrence risk, the intermediate risk category is not associated with clear guidance with regards adjuvant chemotherapy. Throughout posts, this was described as the “up to you zone” (*SueDev April 2017*) or the “grey zone” (*Ursula32, Jan 2017*). *Maeve* (*April 2017)* described that an Onco*type* DX test result of 26 had caused her “more worry than my operations, anxiety and desperation.” Women in 25 discussion threads had received an intermediate result, and of these threads, 13 included contributions from women explicitly asking for input into decision making.

In many of these cases, the score became a powerful and direct representation of their current and possible future experiences of cancer, able to be shared with others within this online environment. Indeed many forum users cited their own results when seeking advice to negotiate the meaning of an intermediate score:

Today the test has not really helped. Basically I'm slap bang in the middle! Score was 21[…] The chemo could help but it's a low percentage. [The oncologist] said I have the overall decision. So hard! I don't want to ever have to regret thinking I should have had it, but statistically it's very small amount of possible help[…] Confused! Any advice most appreciated. SueDev, April 2017

This extract represents a common tension observed in women's posts, with women situated between a resistance to undergoing chemotherapy (particularly where they understood this could be of little benefit), and a felt imperative to avoid cancer recurrence by consenting to further treatment. *SueDev* invokes a notion of future “regret” as shaping her treatment decisions. This accords with language used by patients within existing studies, whereby cancer is positioned as an enemy that patients are responsible for “fighting”,^29^ with “good” patients identified as those who identify and actively manage risks of recurrence.^30^, ^31^ However, for many, this fight entails chemotherapy, a treatment associated culturally and experientially with long‐term side‐effects and suffering.^7^ To overcome some of the difficulties of making choices within these evocative contexts, users placed in the intermediate range described manipulating these numerical signifiers to aid decision making. This included re‐adjusting thresholds, reconstituting risk categories or positioning themselves differently within these to aid decision making:

My score was 23, which is a medium risk but on the lower side of medium. And I would only benefit from chemo another 4% so I don't need it. Iris, July 2015

The other factor influencing my decision was the knowledge that studies have been conducted where the intermediate group was redefined as 11 to 26 which put me firmly into the High category. Bonne, December 2016

Some women with experience of the test advised others to devise personal thresholds prior to receiving results:

It's important you have a cut off point going in….mine was 18‐24 and they would really have to sell me chemo. SunshinePeggs, April 2017

Though depicted as powerful determinants of chemotherapy decision making within some online posts, these examples show that Onco*type* DX results and associated recommendations for chemotherapy were not interpreted so unambiguously by all users. Instead, (anticipated) Onco*type* DX results could be engaged with by patients in varying ways. Interpretations of test scores thus cannot be reduced to their “objective” biomedical significance, but must be understood as shaped by and shaping shared experiences of patient collectives, and the wider meanings of cancer and its treatment.^32^

### 3.3 Attending to uncertainty?

Despite some women attributing the gene expression profiling result with particular authority in shaping treatment choices, others expressed scepticism of the test's ability to aid treatment decision making. Some users noted that despite clinician opinion, statistics and test results, ultimately there “are no crystal balls” (*GrannyG, April 2017*). In some cases, this was linked to the elusive and insidious nature of cancer as a disease, with posts describing “rogue” or “stray” tumour cells. Users’ experiences, including memories of historical treatment, reinforced this sense of the unpredictability of cancer. For example:

Just 2 years after finishing Chemo I was diagnosed with bone [metastases] to my sternum! So a sneaky cell managed to hide from the chemo…..I suppose what I'm saying is Chemo doesn't necessarily give you 100% guarantee! SouthernGirl, Feb 2017

I met two ladies who were back for a recurrence despite having had chemo and radiotherapy, so it seems as if it's just a roll of the dice anyway. Huggy, August 2015

Some women described that ultimately, biomedical knowledge and techniques could not provide a definitive answer as to whether their cancer would recur, and as to whether chemotherapy was an appropriate option. As such, some users discussing Onco*type* DX results articulated that the onus was on themselves to make the final decision with regards chemotherapy. This ultimate uncertainty can be linked to posts emphasizing the ability of cancer to evade detection, with medical techniques unable to confirm whether their cancer had been removed in its entirety. *SouthernGirl* elaborated further:

None of us can see into the future, so we have to make a decision on the information we have.

As we have seen above, the “information we have” went beyond test results and clinical judgement, to indirect experience and expectations of treatment. Embodied and relational elements of decision making following gene expression profiling were also emphasized by forum users. Responding to posts seeking advice with regards chemotherapy choices, users were often encouraged to make decisions that were personal to their circumstances, or based on emotion or embodied experience, by doing what “feels right” *FionaO, Jan 2017*. Reflecting on her negotiation of intermediate category, one user drew on the biological characteristics of her specific tumour type to inform her decision, but ultimately gave authority to personal “feeling”:

I am waiting for my oncotype dx result and have decided that if it is a middling result and I get a say then I will have the chemo as my cancer is grade 3 and an aggressive little thing and because I am a natural worrier so know if I don't have it it will pray on my mind afterwards but everyone is different ‐ in this situation how you feel is more important than statistics. GrannyG, April 2017

Potential *future* emotions were also emphasized, should one refuse chemotherapy only for their cancer to return:

I was in a similar boat last December […] I wanted to take whatever risk reduction I could get so I went with it. I also kept thinking ‘what if I don't’… how would I feel if there was recurrence and I hadn't chosen to go through with chemo? Explorer, March 2016

As we have seen, the emphasis on the personal was informed by cultural narratives and memories of cancer and treatment, and an embodied sense of vulnerability to cancer cells.^33^ In these contexts, women's responses to gene expression profiling test results are therefore not easily predicted; for example, those with a low recurrence score did not always indicate that they would eschew chemotherapy. In what follows, we discuss our findings, and their implications for sociological explorations of treatment decision making in the context of novel biomedical techniques.

## 4 DISCUSSION

Our analysis of online forum discussions has begun to capture how individuals are negotiating gene expression profiling, as they document reflections on this technology in online spaces. Comments from some women positioned Onco*type* DX results as “personalized,” interpreting the information it provides as “tailored” to their cancer, and as superior to existing techniques assisting chemotherapy decision making. This may be shaped by wider discourses of hope and hype surrounding the potential of genomic medicine, highlighted in existing sociological studies [see 2,26]. The quantification of potential recurrence risk as a single score was a particularly powerful characteristic of the test for some women. Onco*type* DX results allowed for the sharing of what was interpreted as *personal* risk, with women disclosing scores in online forums to seek individualized support from others. This accords with existing social scientific research, which has shown that numerical presentations of risk attained through molecular techniques can provide reassurance for individuals post‐treatment [see 32], by increasing certainty and control in the context of disease.

Nevertheless, despite its purported aim to facilitate treatment decision making by predicting recurrence and estimating chemotherapy benefit for individual women, Onco*type* DX testing could be experienced as ambiguous. Other work has shown that the quantification of risk to guide treatment may invoke vulnerability for patients, provoking a sense of foreboding and insecurity by situating individuals in a space between health and illness.^28^ This research has shown some of the ways in which the gene expression profiling result, presented as a singular, numerical representation of recurrence risk, was open to interrogation by women. Users interpreted results in light of more widely adopted techniques used to estimate recurrence risk, such as protein receptor status, and algorithms founded on population data. Understandings articulated by individual women were situated within emotional responses to chemotherapy and cancer in the present, but also possible futures and long‐term consequences of decision making. Through the accounts described above, we have also demonstrated that interpretations of results are informed by sociocultural depictions of cancer and treatment, with chemotherapy and suffering depicted as *necessary* to recover from cancer [see 7,31,34]. Several women discussed the potential for regret at not proceeding to chemotherapy, in some cases linked to perceptions of the disease as insidious, and women's awareness of the inability of biomedical tools to detect “stray” cells.

Decision making was particularly complex where recurrence scores signalled an intermediate risk of cancer recurrence. In these cases, women often appealed to other forum users for guidance on treatment decisions, and most clearly articulated a sense of being positioned between an imperative to treat cancer and cultural narratives of chemotherapy as entailing suffering.^7^ Further, these decisions took place within a context of wider observations of cancer and its recurrence, where the disease was shown to ultimately be unpredictable, and treatment efficacy uncertain. Many women were thus aware of the limitations of gene expression profiling, which remained unable to provide a definitive answer as to whether their cancer would recur, or whether chemotherapy would be able to prevent recurrence. In some cases, women thus encouraged others to privilege experiential or affective knowledge in chemotherapy decision making, over the test score itself [see also 35].

Overall, our analysis has shown that gene expression profiling did not always straightforwardly facilitate decision making with regards proceeding to chemotherapy. This is despite the fact that some forum users represented the test as determining choices. For many women, the test score was not interpreted as clear‐cut, instead results were given meaning and transfigured in light of personal experiences, sociocultural discourses of cancer and chemotherapy, and the limitations of, or expectations for, novel techniques in cancer care. This has implications for the use of genomic prognostic testing within the health service, with clinician‐patient discussions of test scores needing to account for varying interpretations of the meaning of these results, as well as differing and very personal experiences of anxiety surrounding cancer.^33^, ^34^

Online forums were depicted by users as playing a role in women's negotiations of these novel techniques. Accessing and interacting within online spaces further distributes decision making, as treatment options themselves become more diffuse. Patients are today required to make sense of new forms of clinical information and medical techniques, with these experienced by smaller, subgroups of individuals. Women used online forums to document competing treatment options and emotions, to share experiences and to seek advice from others. Online research methods therefore enabled us to observe aspects of decision making as an evolving process distributed amongst a wide range of settings and individuals, enrolling unknown and anonymous others over time and space.^12^, ^13^

Whilst online forums have provided insight into intimate accounts of treatment decision making as shaped by gene expression profiling, their use to access women's experiences does have limitations. We cannot say with certainty that we were able to capture accounts from a diverse group of women, with Internet access likely to reflect wider social and structural inequalities.^36^ This research, however, did not intend to be generalizable in a statistical sense, but to shed light on the breadth and potential complexity of decision making in the context of a novel genomic technique.^37^ The absence of in‐depth narratives, with analysis focusing on short posts which were sometimes devoid of context, has also meant that we were not able to explore wider impacts on decision making in great depth. Further research is required to learn more about how patients seek and share information with others, and the impact of online forum use itself on decision making about cancer treatment. This may offer guidance to patients and clinicians about how online forums might be best used at this difficult time. These issues are being addressed by complementing this online research with ongoing qualitative interviews.

## 5 CONCLUSION

This study of accounts of gene expression profiling has shed light on how women are engaging with and negotiating novel genomic techniques as they become integrated within routine cancer care in the UK, and the resources they draw on in this regard. Importantly for clinical practice, we have shown that the women represented in this research did not always interpret Onco*type* DX scores straightforwardly, with these results taking on varying significance according to factors including personal encounters with cancer, and potential regret for declining treatment. This emphasizes the importance of holistic treatment decision making between patients and clinicians, which may engage with loved ones’ experiences of the disease, “gut feelings,” emotions and anticipated futures.

Online forums have proved to be a valuable resource to explore perceptions of gene expression profiling as articulated by women in the midst of chemotherapy choices. These are also emblematic of the contemporary distribution of decision making, which has the potential to become reconfigured as genomic techniques and “personalized” treatment regimes become further integrated within clinical care. In‐depth qualitative research will provide deeper insight into the emotional and embodied elements of these treatment choices, and their interplay with genomic techniques alongside more established means of informing treatment decisions in cancer care.

## CONFLICT OF INTEREST

The authors have no conflict of interest to declare.

## Note

1 Generally breast cancers that are human epidermal growth factor receptor 2 (HER2) negative, oestrogen receptor (ER) positive and lymph node‐negative.
